# A novel method for the establishment of autologous skin cell suspensions: characterisation of cellular sub-populations, epidermal stem cell content and wound response-enhancing biological properties

**DOI:** 10.3389/fbioe.2024.1386896

**Published:** 2024-04-05

**Authors:** Michael Peake, Chris Dunnill, Khalidah Ibraheem, Adrian Smith, Douglas J. Clarke, Nikolaos T. Georgopoulos

**Affiliations:** ^1^ School of Applied Sciences, University of Huddersfield, Huddersfield, United Kingdom; ^2^ Centre for Dermatology Research, Division of Musculoskeletal and Dermatological Sciences, Faculty of Biology, Medicine and Health, The University of Manchester, NIHR Manchester Biomedical Research Centre, Manchester, United Kingdom; ^3^ Department of General Surgery, Calderdale and Huddersfield NHS Foundation Trust, Huddersfield, United Kingdom; ^4^ Biomolecular Sciences Research Centre, Industry and Innovation Research Institute, Sheffield Hallam University, Sheffield, United Kingdom

**Keywords:** wound healing, cell therapy, autologous transplantation, tissue engineering, reepithelialisation, myofibroblasts, epidermal regeneration, epidermal stem cell population

## Abstract

**Introduction:** Autologous cell suspension (ACS)-based therapy represents a highly promising approach for burns and chronic wounds. However, existing technologies have not achieved the desired clinical success due to several limitations. To overcome practical and cost-associated obstacles of existing ACS methods, we have established a novel methodology for rapid, enzymatic disaggregation of human skin cells and their isolation using a procedure that requires no specialist laboratory instrumentation and is performed at room temperature.

**Methods:** Cells were isolated using enzymatic disaggregation of split-thickness human skin followed by several filtration steps for isolation of cell populations, and cell viability was determined. Individual population recovery was confirmed in appropriate culture medium types, and the presence of epidermal stem cells (EpSCs) within keratinocyte sub-populations was defined by flow cytometry via detection of CD49 and CD71. Positive mediators of wound healing secreted by ACS-derived cultures established on a collagen-based wound-bed mimic were detected by proteome arrays and quantified by ELISA, and the role of such mediators was determined by cell proliferation assays. The effect of ACS-derived conditioned-medium on myofibroblasts was investigated using an *in-vitro* model of myofibroblast differentiation via detection of α-SMA using immunoblotting and immunofluorescence microscopy.

**Results:** Our methodology permitted efficient recovery of keratinocytes, fibroblasts and melanocytes, which remained viable upon long-term culture. ACS-derivatives comprised sub-populations with the CD49-high/CD71-low expression profile known to demarcate EpSCs. Via secretion of mitogenic factors and wound healing-enhancing mediators, the ACS secretome accelerated keratinocyte proliferation and markedly curtailed cytodifferentiation of myofibroblasts, the latter being key mediators of fibrosis and scarring.

**Discussion:** The systematic characterisation of the cell types within our ACS isolates provided evidence for their superior cell viability and the presence of EpSCs that are critical drivers of wound healing. We defined the biological properties of ACS-derived keratinocytes, which include ability to secrete positive mediators of wound healing as well as suppression of myofibroblast cytodifferentiation. Thus, our study provides several lines of evidence that the established ACS isolates comprise highly-viable cell populations which can physically support wound healing and possess biological properties that have the potential to enhance not only the speed but also the quality of wound healing.

## 1 Introduction

Wounds such as those caused by burns, ulcers and physical impact result in a breach of the human skin barrier, subsequently exposing the underlying tissue, which can lead to opportunistic microbial invasion and establishment of infection. The body’s immediate response to skin damage is a complex and dynamic biological process known as wound healing that is driven by the spatially and temporally coordinated action of various cell types and extracellular factors (Dunnill et al., 2017). However, wounds that are either highly extensive (such as burns) or complex and non-healing (such as diabetic ulcers) greatly curtail the effectiveness of clinical wound management, leading to extended patient care thus resulting in enormous financial burden for healthcare providers. For the National Health Service (NHS) in the United Kingdom, the annual financial burden of care for patients with wounds and associated comorbidities in 2012/13 was £5.3 billion, with a 135% increase in the cost of care for patients with unhealed wounds compared to healed wounds (Guest et al., 2017); the cost of wound management is now estimated to have risen to £8.3 billion, of which £2.7 billion and £5.6 billion were associated with managing healed and chronic wounds, respectively (Guest et al., 2020).

Historically, treatment of extensive wounds has utilised split-thickness skin grafting (STSG), whereby an autologous skin graft is taken from the patient and placed onto the wound site, allowing for the donor site to heal. This method of intervention however is relatively outdated and, in some cases, poses caveats including inefficiency in healing, aesthetic displeasure and lengthened patient hospital stays due to large amounts of donor site skin used (Bayat et al., 2003; King et al., 2014; Guest et al., 2017). As a result, there is an urgent clinical need for producing novel, alternative approaches to achieve rapid and efficient recovery in wound care and improvement in the overall wellbeing of patients. Examples of alternative approaches include epidermal grafting which may deliver similar healing rates to STSG, yet only the epidermal layer of skin is harvested, leading to reduced utilisation of donor site resources (Kanapathy et al., 2016; Kanapathy et al., 2021). Another approach involves the use of bioengineered tissue (*in vitro* or 3D bio-printed) whether epidermal, dermal or composite, permanent or temporary skin replacements, these skin substitutes primarily function as barriers for prevention of fluid loss and microbial contamination (Vig et al., 2017; Sörgel et al., 2023). However, bioengineered artificial skin grafts are not yet fully optimized, and are currently limited in their clinical application due to financial and time-consuming constraints in the production process (Przekora, 2020; Sörgel et al., 2023).

Other approaches encompass isolation and expansion of cells (keratinocytes and fibroblasts), to significant quantities and subsequently they can be utilised in various procedures such as allogeneic cell therapy, whereby cells are preserved until required for direct application, or introduced to constructs such as scaffolds (Kirsner et al., 2012; Chen et al., 2023) However, use of allogeneic cells is limited due to immune rejection, and patients may require immunosuppressive drugs in concomitance to the treatment (Jorgensen et al., 2023).

Despite these promising developments, STSG still represents the main method of choice in wound care, because these novel alternative strategies have been unable to contemporaneously offer improvements in cost-effectiveness, ease of practical use (whereby a surgeon or nurse could operate the technology), and/or show similar or considerable effectiveness in accelerating the healing response compared to STSG. One promising type of strategy that has the potential to overcome such obstacles is through a cell-based, “point-of-care” therapy which involves use of autologous (rather than allogeneic) skin cells (Zhao et al., 2016). The principle of this approach involves enzyme-based solutions to isolate viable and proliferative cells from a patient’s own skin *in situ*, which can then be immediately applied to the wound area (Wood et al., 2012; Holmes IV et al., 2018; Manning et al., 2019). An attractive advantage of autologous cell-based therapies is that the formation of an autologous cell suspension (ACS) requires a much smaller amount of donor site skin. Unlike using STSG, which requires donor to wound site ratios of 1:1 or 1:2 (whether meshed or not), ACS application may require remarkably less donor skin, and in some cases it has been suggested that even an 1:80 ratio might be effective (Holmes IV et al., 2018). However, although the concept of autologous cell-based therapy is highly promising, it has not shown adequate clinical success, and the associated cost of currently available devices for ACS isolation remains extremely prohibitive.

To overcome the practical and cost-associated limitations of the currently available laborious methods and expensive devices for preparation of ACS, we have recently established a novel method for rapid, enzymatic disaggregation of human skin cells and their isolation using a patented procedure that requires no specialist laboratory instrumentation and is performed at room temperature. Here, we report for the first time the characterisation of the types of cells isolated and their viability, we provide evidence that the isolated ACS contains epidermal stem cells, and we define the biological properties of the keratinocyte population, which include their ability to secrete positive mediators of wound healing as well as their capacity to modulate myofibroblast cytodifferentiation.

## 2 Materials and methods

### 2.1 Autologous cell suspension (ACS) isolation, determination of cell yields, cell type recovery and maintenance

ACS samples were established using the VeritaCell methodology (http://veritacell.com/). The method (detailed in Supplementary Figure S1) is based on enzymatic disaggregation of split-thickness skin specimens followed by optimised patented filtration-based steps for the isolation of skin cell populations at room temperature within 35–60 min from skin removal (“Method and apparatus for cell isolation and isolated cells for wound healing”, WO2021048441A1). All ACS samples were prepared from skin specimens either: a) collected from the Huddersfield Royal Infirmary through routine abdominal surgical procedures with National Health Service (NHS) Research Ethics Committee (REC) approval (ref no: 15/EM/0265) and informed consent from patients with no history of skin malignancy, or b) acquired commercially from Genoskin (Toulouse, France).

Following completion of the VeritaCell procedure, final cell suspensions were cultured in appropriate cell culture medium. For recovery and establishment of normal human epidermal keratinocyte (NHEK) populations, the ACS cultures were maintained in ‘complete’ (supplemented with Human Keratinocyte Growth Supplement, HKGS) Epilife™ (#MEPI500CA) medium (Thermo Fisher Scientific, Loughborough, United Kingdom) in T25 cm^2^ cell culture flasks (Sarstedt, Leicester, United Kingdom) pre-coated with 200 mg/mL human Collagen IV (Merck, Watford, United Kingdom). For recovery of human dermal fibroblasts (HDFs), ACS cultures were grown in DMEM with 10% fetal bovine serum (FBS). For isolation of melanocytes, ACS cultures were kept in Epilife™ until the first passage, before cultures were switched to Melanocyte growth medium (MGM)-4 SingleQuots™ (Lonza, Switzerland). In some experiments, the HaCaTa cell line was used, the establishment of which and its culture conditions have been described elsewhere (Al-Tameemi et al., 2014). An EVOS^®^ XL Core microscope (Thermo Fisher) was used to routinely image cells and to obtain images of cell cultures at different time-points post-establishment. To determine yields of viable skin cells immediately after isolation and at specific time-points post initial culture, cell counts were performed using a haemocytometer and Trypan blue (0.4% w/v) staining.

### 2.2 Cell proliferation assays

Quantitative detection of NHEK and HaCaTa cell proliferation was performed using the CellTiter 96^®^ AQueous One Solution Cell Proliferation Assay (Promega, Southampton, United Kingdom), as recommended by the manufacturer and absorbance determined by spectrophotometric measurements at 492 nm using a FLUOstar OPTIMA (BMG Labtech, Bucks, United Kingdom). Following normalisation using medium-only ‘blank’ wells, cell proliferation was calculated as described previously (Dunnill et al., 2020).

### 2.3 Flow cytometry

Cells from freshly isolated autologous suspensions were aliquoted at 100,000 cells/mL in 1.5 mL tubes and subsequently labelled with either FITC-conjugated CD71 (MCA1148FT, Bio-Rad, Watford, UK) and PE-conjugated CD49 (MCA1457PE, Bio-Rad) primary antibodies, or with FITC- (553565, BD Biosciences) and PE-conjugated (556650, BD Biosciences) isotype-matched control IgG. Cell suspensions were incubated with test and control antibody for ∼1 h at 4°C, before centrifugation at 300 g for 5 min and removal of media. Cells were then rinsed twice in PBS +1% FBS buffer. For surface protein expression, immunolabelled cells were acquired on a Guava EasyCyte 5 flow cytometer and data analyzed using EasyCyte software (Millipore, Watford, United Kingdom) as previously (Ibraheem et al., 2022).

### 2.4 Proteome arrays

Cells from ACS were maintained in culture for up to 7 days with daily medium-change of the Epilife™ medium either supplemented (‘complete’) or non-supplemented with HKGS (see Section 2.1). On days when the ACS culture medium was to be collected, cells were always incubated in non-supplemented Epilife™ medium for 24 h prior to collection, and this was denoted as ACS conditioned medium (CM). Detection of analytes released *in vitro* from collected conditioned medium (at the indicated time points following culture initiation) from ACS-derived cultures was performed using an angiogenesis array kit (#ARY007) and a cytokine array kit (#ARY005B) (both from R&D Systems, supplied by Bio-Techne, Abingdon, United Kingdom). Array processing was carried out following the manufacturer’s instructions, with the exception of the supplied streptavidin secondary antibody being replaced by IRDye^®^ 800CW Streptavidin antibody (#926–32230, LI-COR Biosciences, Cambs, United Kingdom), to enable fluorescence-based detection on an Odyssey™ Infra-red Imaging system (LI-COR), as detailed (Ibraheem et al., 2018).

### 2.5 ELISA assays

Enzyme-linked immunosorbent assay (ELISA) was used for quantitative determination of IL-1α (#DLA50, R&D Systems, Bio-Techne), HMGB1 (#NBP2-62766, Novus Biologicals, supplied by Bio-Techne) and HSP90α (#NBP2-76448, Novus Biologicals) in ACS conditioned medium. Protocols for all ELISAs adhered to the manufacturer’s instructions and data was obtained using a FLUOstar OPTIMA plate reader by spectrophotometric detection at 450 nm as previously (Dunnill et al., 2020).

### 2.6 Western blotting

Quantitative protein detection was performed by immunoblotting as detailed elsewhere (Ibraheem et al., 2022). Briefly, cell cultures were lysed and lysates established using an SDS-based buffer (Georgopoulos et al., 2014) containing 2 mg/mL DTT and 1% (v/v) protease inhibitor cocktail set 3 (Calbiochem, supplied my Merck). Following SDS-PAGE electrophoresis using 10-well NuPAGE™ gels (#NP0321, Invitrogen, Merck, United Kingdom), proteins were transferred onto Immobilon-FL PVDF membrane (#IPFL00010, Merck Millipore) in an Xcell II blot module (Invitrogen). Membranes were incubated with anti-alpha smooth muscle actin (α-SMA) antibody (A2547, Sigma-Aldrich, supplied by Merck) at 1:2000 dilution overnight at 4°C, followed by Alexa Fluor^®^ 680 Goat anti-mouse IgG (A21057, Invitrogen, Merck) at 1:10000 dilution. Antibody specificity was controlled as explained in full detail elsewhere (Ibraheem et al., 2018). Membranes were subsequently scanned on an Odyssey™ system and densitometry performed using Image Studio software version 5.2 (LI-COR).

### 2.7 Immunofluorescence microscopy

HDFs were seeded onto Teflon-coated 12-well glass slides as detailed previously (Georgopoulos et al., 2014) at 980 cells/well (50 cells/mm^2^) and they were cultured for up to 7 days. Culture conditions involved an alternating pattern of culture in DMEM +10% FBS, followed by culture the day after with medium alone or medium containing TGF-β (Sigma-Aldrich) at 2 ng/mL; the medium was either Epilife™ (control) or CM and the exact procedure followed is explained in full detail and justified in the Results (Section 3.4). Cells were then fixed with 10% Formalin solution for 15 min at room temperature and permeabilized using a solution containing PBS +0.5% Triton-X + 10% goat serum for 1 h at room temperature. HDFs cells were then dual-labelled with, firstly, anti-α-SMA antibody (A2547, Sigma-Aldrich) at 1:400 dilution and secondly, anti-vimentin antibody (D2H13, Cell Signalling Technology) at 1:100 dilution in a solution containing Tris-buffered saline (TBS) + 0.1% (v/v) sodium azide +0.1% (v/v) BSA and incubated overnight at 4°C. The next day, cells were incubated with Goat anti-Mouse IgG (H + L) cross-adsorbed antibody Alexa Fluor 488 (A-11001) and Goat anti-Rabbit IgG (H + L) cross-adsorbed antibody Alexa Fluor 594 (A-11012) (both from Thermo Fisher) at 1:100 and 1:1000 dilutions, respectively, for 1 h at room temperature. Slides were counterstained with Hoechst 33258 (Thermo Fisher) at 0.1 mg/mL in PBS and mounted with anti-fade solution, as detailed elsewhere (Georgopoulos et al., 2014). Slides were visualised using a Zeiss Axio Imager Z1 and images captured with Zeiss AxioCam MRm Rev.3 digital camera, using the same exposure times for all slides. Images were analysed using ZEN (Carl Zeiss Ltd., Herts, United Kingdom) and ImageJ software (https://imagej.net/).

### 2.8 Statistical analysis

All graphs were prepared using GraphPad Prism 6 version 6.0.0 for Windows (GraphPad Software, Boston, Massachusetts USA, www.graphpad.com). For statistical analysis of multiple data sets, one-way ANOVA was employed with Dunnett’s multiple comparison test. Statistical significance is indicated appropriately (denoted by defined asterisks) in each figure caption.

## 3 Results

### 3.1 Establishment of ACS using the VeritaCell methodology, determination of viable cell yields and isolation of different skin cell sub-types

The overarching aim of our methodology for establishment of ACS from human skin was the ability to process split-thickness skin grafts within <1 h (routinely performed within ∼35–60 min) at room temperature, with no specialist equipment or scientific expertise required, with the aim for this to be carried out *in situ* (in a clinical setting) for direct application in patients. As detailed in the Methods, we developed an isolation method that involves recombinant protease-based enzymatic treatment, following which a novel filtration step permits the isolation of human skin cell populations.

**FIGURE 1:**
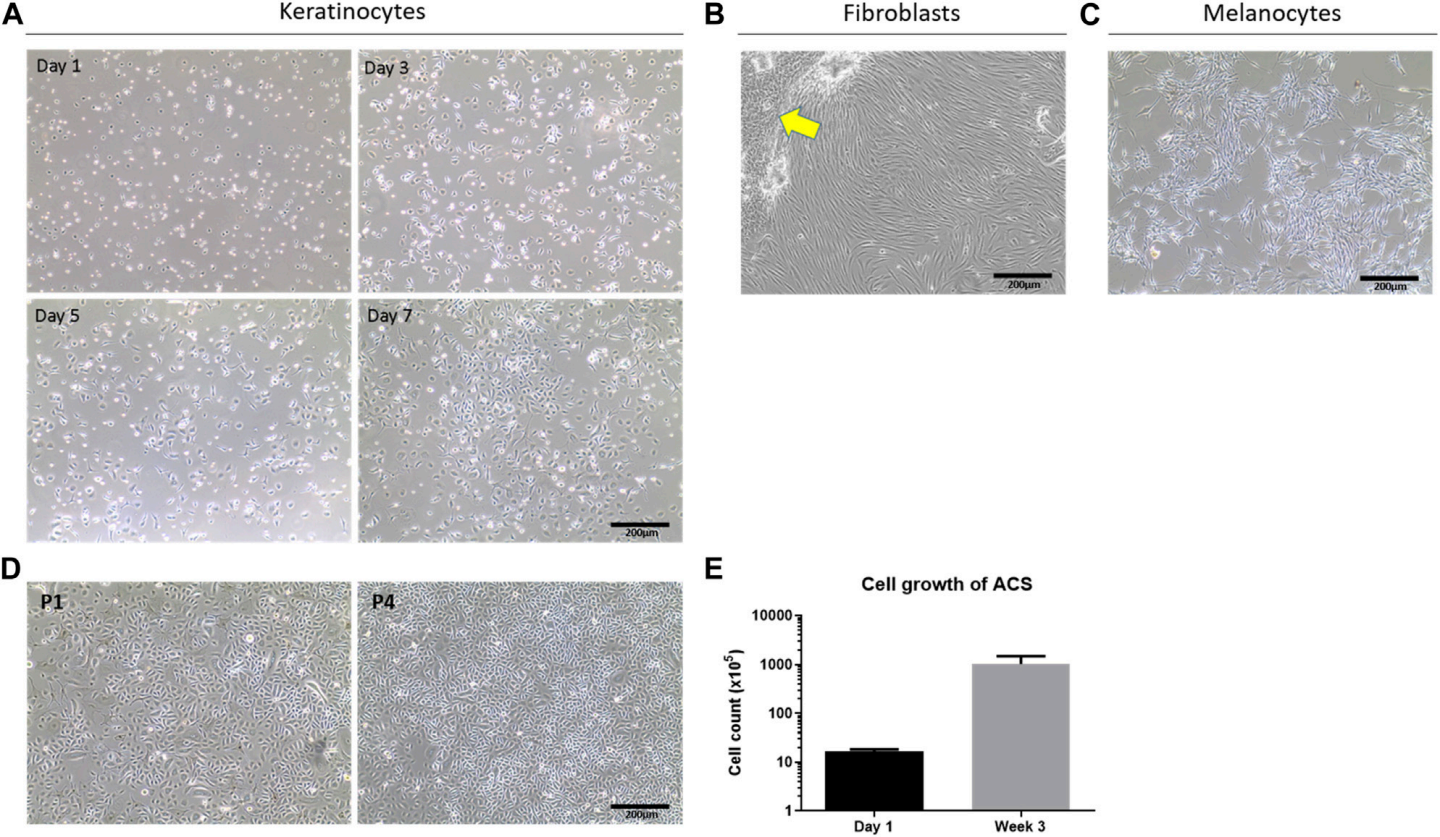
Determination of cell sub-types in ACS isolates and their viability. **(A)** Phase contrast cell imaging time-course of isolated NHEKs from ACS at the indicated time points (Day 1 to Day 7). **(B)** Images of fibroblast (HDF) cultures from ACS isolates in serum-containing medium (arrow highlights remaining NHEK populations displaying features of cytodifferentiation). **(C)** Images of melanocyte cultures established from ACS. **(D)** Examples of long-term NHEK cultures from the first passage (panel P1) to four passages (panel P4). **(E)** Mean cell counts of ACS established from 3 independent skin biopsies (∼3–4 cm^2^) from Day 1 post-isolation, and after 3 weeks of *in vitro* culture. Scale bar: 200 μm.

To study the properties of these autologous cell isolates at different time points *in vitro*, we seeded the recovered cell populations on collagen IV-coated plasticware as an artificial ‘wound bed’ mimic. Provided the skin specimen used was processed within <24 h post-operatively, our methodology consistently resulted in the isolation of a high number of viable cells, which was 5 ± 0.1 × 10^5^ cells per cm^2^ of tissue (Figure 1E). When the cells were cultured on such a wound-bed mimic in serum-free Epilife medium, they rapidly adopted a normal human epidermal keratinocyte (NHEK) morphology and, following an initial lag phase (Days 1–3), rapidly reached high confluency within 1 week in culture (Figure 1A). Moreover, these cells demonstrated the capacity for sustained *in vitro* proliferation for several passages (∼4), and over 3 weeks exhibited an >60-fold increase in cell numbers (Figure 1D, E). Notably, both our cell yields and observations on cell behaviour were strikingly similar to those achieved using previously reported, optimal methodologies for the establishment of keratinocytes by overnight dispase treatment and subsequent trypsin-based isolation procedures (Aasen and Izpisua Belmonte, 2010).

As successful wound healing does not only depend on epidermal layer re-population by keratinocytes but also requires the action of fibroblasts to support the wound response as well as melanocytes for skin re-pigmentation, we investigated whether the ACS isolates also comprised such cell populations. Using selective cell culture medium types (as detailed in the Methods) we confirmed, by morphological assessment, the presence of normal human fibroblasts (HDFs) upon culture in serum containing DMEM medium, and melanocytes upon culture in MGM medium as shown in Figure 1B, C, respectively.

Overall, these findings provide evidence that the VeritaCell methodology permits efficient recovery of not only keratinocytes but also fibroblasts and melanocytes, it allows rapid isolation at room temperature of high numbers of viable cells which remain viable upon long-term culture, and thus the method completely negates elaborate cell culture procedures.

### 3.2 The presence of epidermal stem cells (EpSCs) in ACS isolates

Epidermal stem cells (EpSCs) are multipotent adult stem cells which reside in the basal layer of the skin and the bulge region of the hair follicle, and are important in epithelial homeostasis, as well as in tissue regeneration and restoration of the epidermal barrier following injury. Thus, it was critical to investigate whether the ACS isolates contained such EpSCs sub-populations. Although an unequivocal marker for EpSCs characterisation remains elusive, previous investigations have identified a sub-population of cells expressing a combination of CD49 (α6-integrin) and CD71 (transferrin receptor) proteins, which allowed them to demarcate cells with a putative basal stem cell-like phenotype based on a defined CD49^high^/CD71^low^ expression pattern (Webb et al., 2004).

**FIGURE 2:**
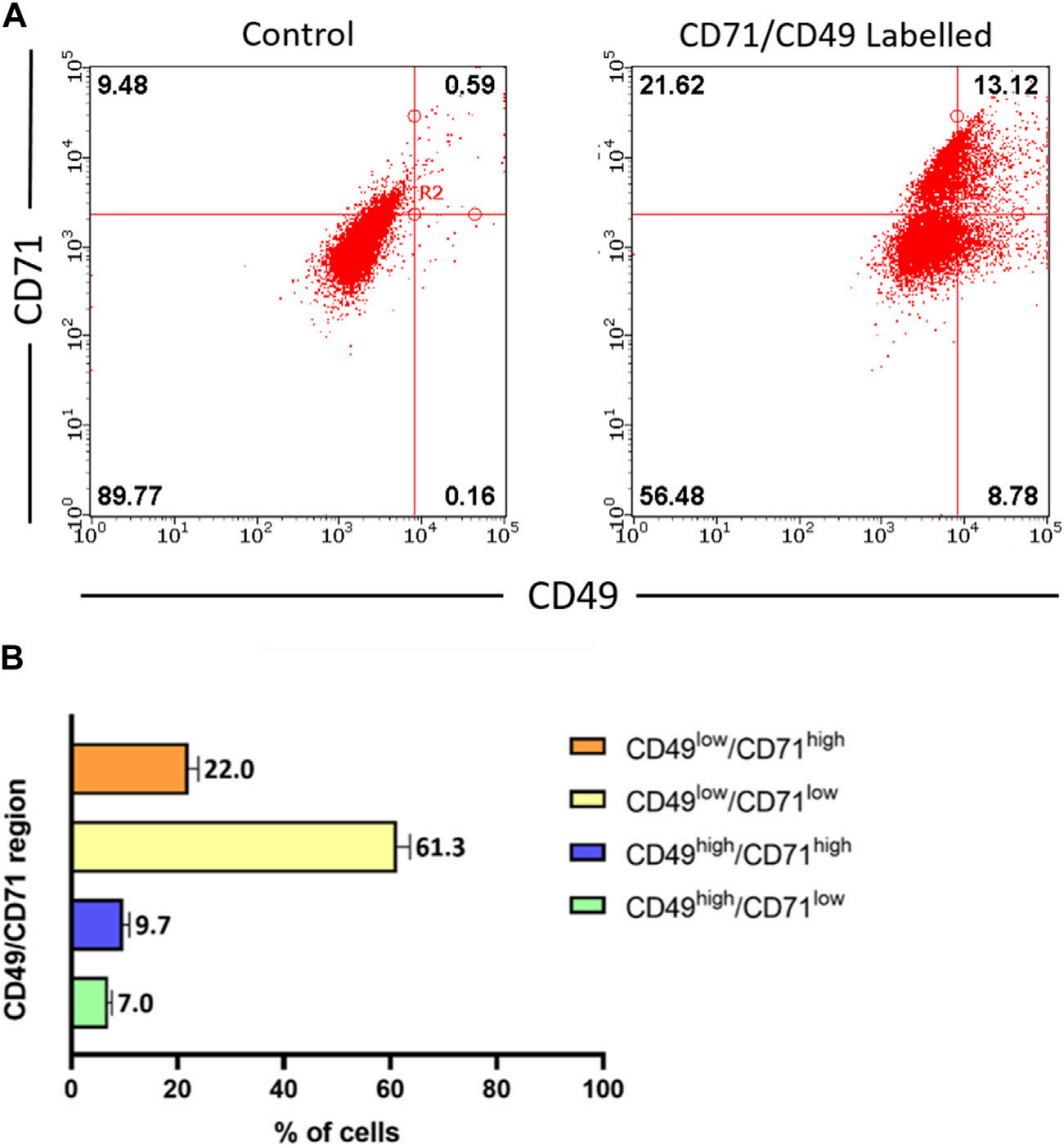
Characterisation of stem cell populations present in freshly isolated ACS. ACS cell populations were dual-labelled with FITC-conjugated anti-CD71 and PE-conjugated anti-CD49 antibodies (alongside FITC- and PE-conjugated isotype control antibodies) and analysed by flow cytometry. **(A)** Representative fluorescence intensity dual dot plots for detection of CD71 (y-axis) and CD49 (x-axis) protein expression (right panel). Quadrants and regions were established using isotype control antibodies to determine the parameters for background CD71/CD49 positivity (left panel). **(B)** Summary of results for CD71 and CD49 expression patterns from independent ACS isolates (n = 3).

Using multi-colour flow cytometry analysis, we determined the expression of both CD49 and CD71 in freshly isolated ACS (Figure 2A). Analysis of independent samples (n = 4) indicated the consistent presence of a small cell sub-population expressing exhibiting CD49^high^ and CD71^low^ expression, which accounted for ∼7% of the total ACS population (Figure 2B). These experiments confirmed that our autologous suspensions included the previously characterised basal, stem cell CD49^high^/CD71^low^ sub-population, which has been previously characterised as the sub-population that drives the regenerative potential of epidermal cells *in vitro* and *in vivo* (Webb et al., 2004; Metral et al., 2017).

### 3.3 Proteomic analysis of the ACS secretome reveals sustained release of positive mediators of cell proliferation and wound healing

**FIGURE 3:**
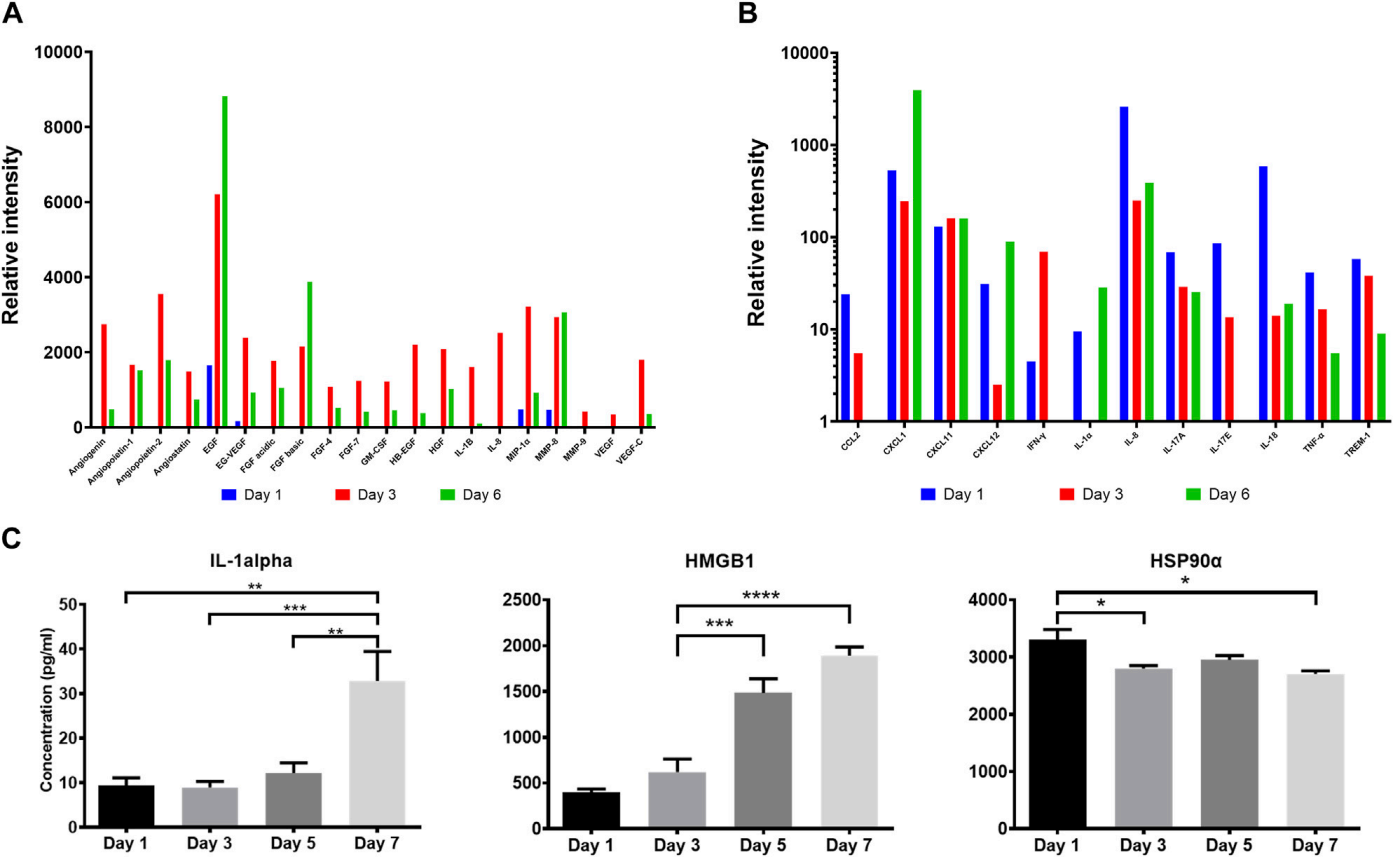
Proteomic analysis of the ACS secretome. Semiquantitative analysis using proteome arrays and quantitative detection by ELISA assays of soluble mediators secreted by ACS-derived cell populations cultured in serum-free medium at the indicated time points. Supernatants from cultures established from ACS were collected and analysed as detailed in the Methods. **(A)** Relative intensity values of densitometric analysis of ACS-secreted soluble factors using an angiogenesis array on days 1, 3 and 6 after isolation. **(B)** Relative intensity values of similar analysis using a cytokine proteome array on the same time-points post-isolation. **(C)** Detection of Interleukin-1 alpha, HMGB1 and Hsp90α in the ACS secretome by analyte-specific ELISA. *, *p* < 0.05; **, *p* < 0.01; ***, *p* < 0.001; ****, *p* < 0.0001; ns, non-significance.

It has been postulated that following their application on wounds, cells in ACS successfully integrate (“take”) into the wound site (Sood et al., 2015; Domaszewska-Szostek et al., 2021), and during this process the incorporated cells may secrete mediators (growth factors and cytokines) for neighbouring cells in the wound to accelerate healing. We therefore screened the ACS “secretome” by employing a proteome array approach. We found that ACS-derived cultures attached to a wound bed mimic (and maintained in serum-free medium) released wound healing-accelerating pro-angiogenic proteins and pro-inflammatory cytokines over 6 days (full results are provided in Supplementary Figure S2). The data for a subset of these secreted factors is presented in Figure 3 and some well-established positive mediators of wound healing include: EGF, FGF, VEGF, CXCL1 and IL-1α (Figures 3A, B). Following initial screening, we used ELISA-based detection to provide a more quantitative analysis; for instance, we demonstrated increasing secretion of IL-1α, with a highly significant concentration at Day 7 of culture (Figure 3C).

Damage-associated molecular pattern (DAMP) proteins, and HMGB1 and HSP90α in particular, are pro-inflammatory proteins released from dying cells and not only modulate innate immune pathways but are also associated with promoting ‘wound-edge’ keratinocyte proliferation and motility (Li et al., 2007; Ranzato et al., 2009; Li et al., 2012). As such mediators could not be detectable by our proteome array approach, we used ELISA analysis and demonstrated the presence of these two critical DAMPs within the ACS secretome, with distinct concentration patterns evident. We observed a steady, gradual rise of HMGB1 levels during culture, while HSP90α displayed its highest concentration on Day 1 and its levels remained sustained throughout (Figure 3C). These findings support the notion that cells derived from our ACS preparations progressively secrete a variety of chemokines and cytokines as well as DAMPs that are well-established enhancers of the process of wound healing.

**FIGURE 4:**
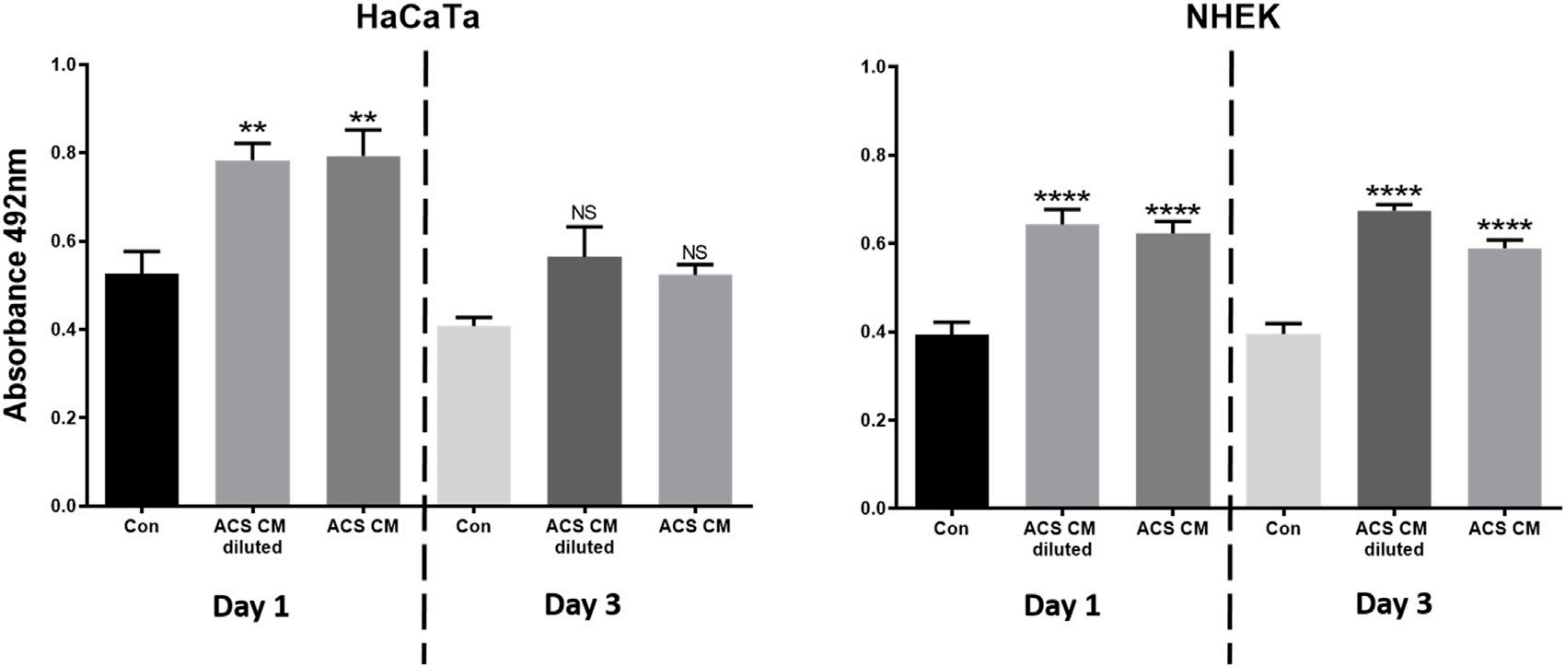
The effect of the ACS secretome on HaCaTa and NHEK cell proliferation. HaCaTa (4 × 10^4^) and NHEK (5 × 10^4^) cells per well were seeded in 96-well plates in Epilife™ complete medium and incubated overnight. Cells were either treated with neat ACS conditioned medium (ACS CM) as well as CM diluted at 1:1 (v/v) ratio with Epilife™, or Epilife™ medium alone (control). The ACS CM used was collected at Day 1 or Day 3 post-ACS isolation. Cell proliferation was determined 72 h later using the CellTiter 96^®^ Aqueous One solution. Results are presented as mean absorbance readings for immortalised HaCaTa cells and primary NHEK cell cultures (bars denote n = 6 technical replicates for a representative experiment). *, *p* < 0.05; **, *p* < 0.01; ***, *p* < 0.001; ****, *p* < 0.0001; ns, non-significance.

We also collected ACS-conditioned medium (ACS CM) at different time points of culture (Days 1 and 3), which was subsequently added directly to cultures of either primary keratinocytes (NHEK) or our previously characterised immortalised ‘adapted HaCaT’ (HaCaTa) cell line (Dunnill et al., 2020). We found that CM from ‘early’ cultures (Day 1) and cells cultured for longer periods (Day 3) markedly enhanced cell proliferation in both immortalised and primary keratinocytes (Figure 4). Interestingly, we observed no differences between the effects of ACS CM and CM that was diluted (1:1 v/v), whilst the effects of the ACS secretome (either neat or diluted) were more prominent in primary (NHEK) keratinocytes in comparison to immortalised (HaCaTa) cells. Therefore, via the ability of the ACS-derived cells to secrete a large panel of wound healing-enhancing mediators of which many are mitogenic factors, the ACS secretome directly enhances keratinocyte proliferation.

### 3.4 Effects of conditioned medium from ACS-derived cultures on myofibroblast cytodifferentiation

**FIGURE 5:**
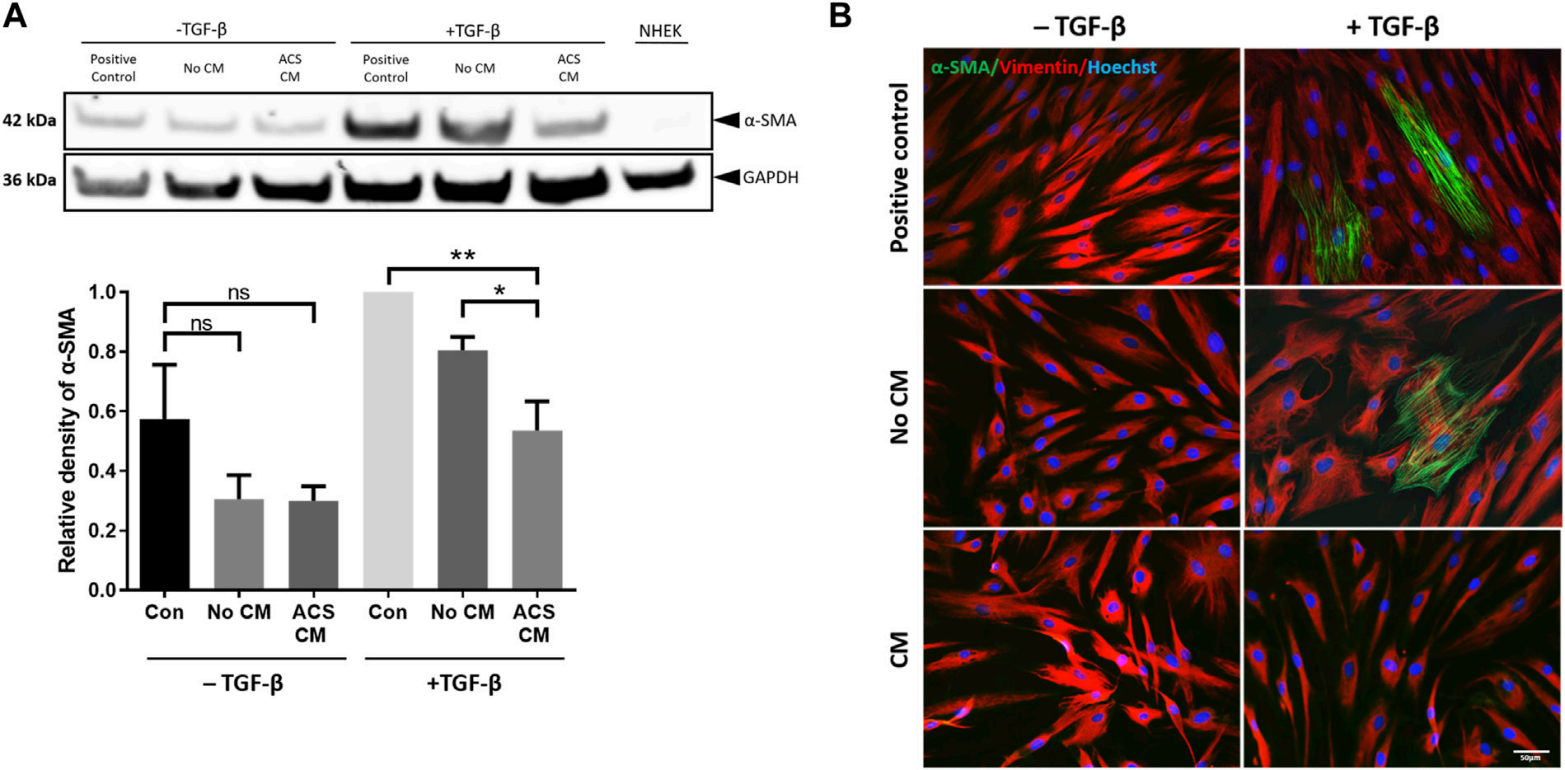
CM from ACS-derived cultures suppresses myofibroblast cytodifferentiation. CM was collected from Day 3 cultures established using ACS isolates as detailed in the Methods. HDFs were cultured at low seeding density (50 cells/mm^2^) under the indicated conditions in 6-well plates for 7 days, after which the cultures were used for preparation of cell lysates (as detailed in the Methods). The different culture conditions and treatments are denoted as detailed in the main text, with two sets of treatment, i.e., presence or absence of TGF-β (“−/+ TGF-β” conditions), whilst NHEK cultures alone served as negative control. **(A)** Representative immunoblotting experiment of detection of α-SMA expression in HDFs treated as indicated (upper panels) and summary of densitometric analysis of relative α-SMA expression for independent experiments (n = 3). *, *p* < 0.05; **, *p* < 0.01; ns, non-significance. **(B)** Detection of α-SMA expression by immunofluorescence microscopy in HDFs cultures of the same density and for the same treatment conditions as in **(A)**. In addition to α-SMA, vimentin expression was also detected, whilst nuclei were visualised by labelling with Hoechst as indicated. Scale bar: 50 μm.

Human dermal fibroblasts (HDFs) play several critical roles during wound healing, including ECM production to support growth of other cell types, but they can also differentiate to myofibroblasts which are characterised by the high expression of smooth muscle actin (α-SMA). Although during a healthy, acute wound response myofibroblasts ultimately undergo apoptosis, inappropriately sustained presence of these cells is associated with dysfunctional tissue repair, excess wound contraction and fibrosis (scarring) (Penn et al., 2012; Darby et al., 2014). Having demonstrated the ability of ACS-derived cells to secrete (immediately after attachment) positive mediators of wound healing as well as mitogenic factors that enhanced keratinocyte proliferation, we investigated whether the ACS secretome may also influence myofibroblast homeostasis. For this purpose, we utilised a previously optimised *in vitro* model of myofibroblast differentiation, which involved low-density seeding and subsequent culture of HDFs in the presence of TGF-β, conditions that trigger cytodifferentiation and α-SMA induction, as detailed elsewhere (Masur et al., 1996). Immunoblotting confirmed the successful emergence of myofibroblasts in such HDF cultures by the detected moderate reduction in expression of the mesenchymal marker vimentin (not shown) and the marked increase in α-SMA protein levels (Figure 5A lanes denoted “positive control” for −/+ TGF-β conditions).

We then examined the influence of the ACS secretome on myofibroblast differentiation by treatment with ACS culture-derived conditioned medium. For this purpose, it was essential to include all appropriate controls to permit accurate direct comparisons to be made between conditions. Thus, in addition to a) control (“Positive control” which involved culture in DMEM medium with 1% FBS), HDFs were treated with b) serum-free Epilife™ medium devoid of any supplements to prevent erroneous provision of exogenous mitogenic factors (such as EGF) contained in the manufacturer’s supplements (condition denoted “No CM”), however 1% FBS was also added similarly to the “positive control”; and c) conditioned medium from ACS-derived cultures maintained in supplement-free Epilife™ for 3 days (condition denoted “ACS CM”), also with 1% FBS. Moreover, because Epilife™ medium contains much lower concentrations of Ca^2+^ (60 μM) than does DMEM (∼2 mM) and calcium levels can significantly influence skin cytodifferentiation, the Epilife™ medium used for these conditions was appropriately supplemented with CaCl_2_. Additionally, during the culture of HDFs the aforementioned medium conditions were employed on alternate days. On the intervening days, all HDFs were cultured in DMEM with 10% FBS in order to support cell viability. This alternating pattern of daily medium changes was maintained for 7 days during the culture period.

When assessed by immunoblotting, although treatment with TGF-β resulted in marked upregulation of α-SMA expression, strikingly the presence of ACS CM suppressed the induction of α-SMA (Figure 5A—upper panels), with protein expression returning to nearly basal levels (i.e., those observed in non TGF-β treated cultures). By contrast, the equivalent control cultures (No CM) exhibited moderate reduction in α-SMA expression. The significance of these findings was confirmed for a series of ACS specimens (n = 3) by using quantitative densitometric analysis (Figure 5A—lower panels). Moreover, we reinforced these intriguing findings by immunofluorescence microscopy studies. Following a series of treatments in the same culture conditions, we visually confirmed the effects of TGF-β as it induced the emergence of myofibroblasts with highly expressed cytoskeletal filament-localised α-SMA protein (Figure 5B). Concordantly with our immunoblotting results, ACS CM suppressed the induction of α-SMA expression in comparison to controls (No CM). Collectively, these findings provided compelling evidence that the ACS secretome not only enhances cellular proliferation but may also attenuate the process of myofibroblasts cytodifferentiation.

## 4 Discussion

The process of skin re-epithelialisation during wound healing is facilitated by rapid proliferation and migration of keratinocytes; failure or deceleration of this step in the healing process is associated with delayed tissue repair, infection and/or chronic wound development. There is increasing evidence that autologous cell suspension-based therapies represent a highly promising approach (Zhao et al., 2016) for burns and various type of chronic wounds (Shukla et al., 2010; Hu et al., 2015; Hayes et al., 2020) and with increasing clinical success (Holmes IV et al., 2018; Kowal et al., 2019). It has been postulated that ACS-associated cells successfully integrate (“take”) into a wound site (Sood et al., 2015; Domaszewska-Szostek et al., 2021) which accelerates re-epithelisation and wound healing, reduce the risk of infection and suppresses discomfort (pain). However, despite the great promise of the approach, the existing technologies for the application of ACS (in the form of ‘spray-on’ skin via a syringe-like applicator or a more sophisticated ‘spraying gun’) have not always achieved the desired clinical promise due to a number of limitations such as high cost *versus* clinical benefit (Manning et al., 2022) whilst obtaining optimal cell populations often requires electrical and/or laboratory instrumentation (Esteban-Vives et al., 2015; Esteban-Vives et al., 2016). Therefore, in light of this clinical promise, it is vital that improved approaches address these limitations whilst ensuring that the established ACS cell populations have all the desired biological properties to enhance wound closure as well as healing ‘quality’, i.e., to restore pigmentation and to ideally suppress scarring.

Our aim is to transform existing ACS technologies for rapid preparation of autologous cell populations in solutions that contain highly-viable cell populations, These can be applied directly and immediately (without culturing) upon isolation in the operating theatre in order to accelerate wound healing. Here we have provided evidence that our novel method permits the rapid establishment of autologous cell suspensions at room temperature and without the use of specialist instrumentation, which: a) include viable keratinocytes, fibroblasts and skin pigmentation-associated melanocytes as well as epidermal stem cell populations, whilst b) the ACS-derived cells possess wound-beneficial biological properties, such as the ability to release positive mediators of wound healing, ability to enhance human keratinocyte proliferation, whilst suppressing the overactivation of myofibroblasts.

Our study has confirmed that the ACS isolates comprise highly viable population of keratinocytes, fibroblasts and melanocytes. Rheinwald and Green first originally described methods for isolation and cultivation of viable keratinocytes from skin, whilst demonstrating the importance of fibroblast ‘feeder’ layers to support keratinocyte growth (Rheinwald and Green, 1975). These feeders aim to support growth by secreting growth factors to the keratinocytes, whilst producing ECM components to facilitate keratinocyte adherence to the culture substratum (Tracy et al., 2016). Using a Collagen IV-coated substratum, we found that this wound-bed mimic was adequate to support rapid recovery and proliferation of keratinocytes in the absence of feeders, which confirmed the quality of the isolates (i.e., high level of ACS cell viability). It is also noted that our filtration method for isolation of skin sub-populations permitted the removal of excess extracellular matrix (ECM) proteins/fragments which was important in the initial attachment of cells (particularly keratinocytes). Critically, without removal of such ECM fragments we observed that cell attachment to the collagen substratum was significantly compromised, which notably curtailed adherence and thus cell recovery (data not shown).

A previous report on the establishment of autologous cell suspensions has provided evidence for recovery of 1.7 × 10^6^ cells per cm^2^ of human skin with ∼75% viability (Wood et al., 2012); notably, the report involved use of technology that utilises specialist electrical instrumentation and requires incubation (heating) of the skin specimens. Here, we report cell recovery of ∼5 × 10^5^ cells per cm^2^ tissue with an ∼85% viability at the point of isolation. Importantly, however, we note that our cell yields and observations on cell behaviour (particularly speed of reaching culture confluency) are in complete agreement with the previously reported, optimal methodologies for establishment of keratinocytes, which represent the ‘gold-standard’ method for human skin processing and keratinocyte isolation. Yet, such optimal procedures involve overnight dispase treatment and subsequent trypsin-based isolation procedures, which requires use of cell culture facilities and specialist instrumentation (Aasen and Izpisua Belmonte, 2010).

Stratum corneum is the outermost epidermal sub-layer represented by a keratinized barrier that is formed by terminally differentiated keratinocytes, which are continually replenished by epidermal stem cells (EpSCs) and transit amplifying cells (TA) residing in the basal layer of the epidermis. EpSCs are hypothesised to be “intrinsically ageing resistant” (Stern and Bickenbach, 2007; Giangreco et al., 2008) and are important not only in adult skin homeostasis, but also in restoring the epidermal barrier after injury (Blanpain and Fuchs, 2006). Epidermal cells in the supra-basal layer have also been shown to de-differentiate and revert to stem cells upon wounding, further supporting the role of the stem cell phenotype in tissue repair (Fu et al., 2001; Mannik et al., 2010) thus substantial skin repair is inextricably linked with the presence of these EpSCs. However, to date, no definite singular marker for EpSCs has been identified and a combination of markers provides a sub-population that shows stem-like properties and functionality. This contrasts to the hair follicle, whereby proteins such as CD200 are used to specifically mark stem cells of the bulge (Purba et al., 2017). Postulations for putative EpSC markers are ongoing, with combinations of cell surface expression of α6-integrin and CD71 being the most extensively characterised. Integrin α6 (also known as CD49), is a cell-surface protein exclusively expressed on the basal cells of the epidermis, which marks both stem and TA cells (Webb et al., 2004). CD71 (transferrin receptor) is highly expressed in actively cycling cells, yet it is expressed at low levels in long-term proliferating neonatal foreskin cells as well as cells in the human hair follicle bulge region (Tani et al., 2000; Webb et al., 2004). It has been reported that the α6^high^/CD71^low^ expression pattern demarcates cells with stem cell-like properties, while α6^high^/CD71^high^ represent TA cells. Importantly, cells with the α6^high^/CD71^low^ phenotype exhibit more potent epidermal renewal capacity (Webb et al., 2004; Metral et al., 2017). In support of these observations, the α6^high^/CD71^low^ phenotype also seems to define a stem cell population in other types of epithelia, such as oesophageal and corneal (Croagh et al., 2007; Hayashi et al., 2008). Here, we have shown that the ACS isolates comprise cells with the α6^high^/CD71^low^ expression profile and a population % that is in accordance with previous observations for the type of skin we used, in our studies mainly abdominal skin samples (Metral et al., 2017). Notably, we observed the same α6^high^/CD71^low^ expression profile whether ACS cells were immuno-labelled directly after isolation or following initial overnight attachment and recovery (not shown). Although we did not specifically isolate this cell sub-population for further assessment of self-renewal capacity and clonogenicity, we suggest that the presence of these cells would be supportive of the high proliferative capacity of our ACS-derived cells based on observations by Webb et al. (2004) and Metral et al. (2017) that the CD49/CD71 EpSC sub-population drives epidermal regeneration *in vitro* and *in vivo*; thus the presence of such cells confirms the ‘quality’ of our ACS isolation procedure.

Moreover, as the aim is to use ACS isolates from a patient’s own skin *in situ* immediately to accelerate wound healing, the presence of the EpSC population could be critical in the ability of ACS to enhance wound healing and it is significant, in line with the strong promise of stem cell-based therapies for cutaneous wound healing (Kosaric et al., 2019).

Our study has demonstrated that immediately upon establishment and attachment to a wound bed mimic, the ACS cell populations exhibited biological properties that can enhance the wound response by providing beneficial extracellular mediators to the surrounding wound bed. We found that the ACS secretome contains a large panel of growth factors including EGF and FGF, which are important for keratinocyte proliferation and motility (Werner and Grose, 2003; Park et al., 2018) as well as containing various cytokines and chemokines which are well-characterised promoters of keratinocyte proliferation and motility (Maas-Szabowski et al., 1999; Spiekstra et al., 2007; Kroeze et al., 2012), with pro-inflammatory cytokine IL-1α prominently over-expressed in the ACS secretome. We also detected more recently established positive mediators which are not attributed to viable cells but can be released from perishing (non-viable) cells via processing and isolation procedures to establish ACS from skin. Damage-associated molecular pattern (DAMP) proteins, and HMGB1 and HSP90α in particular, are molecules released from dying cells that not only promote innate immune pathways but are also associated with promoting ‘wound-edge’ keratinocyte proliferation and motility (Li et al., 2007; Ranzato et al., 2009; Li et al., 2012; Bhatia et al., 2016; Bhatia et al., 2018). We observed a steady and gradual rise of HMGB1 levels during culture, while HSP90α displayed its highest concentration on the first day of culture and its levels remained sustained throughout. The prompt detection (<24 h of culture) suggests that administered ACS may have an immediate effect on ‘wound edge’ keratinocytes whilst also releasing beneficial soluble factors that can enhance wound healing.

We have also provided evidence that at least some of the components of the ACS secretome are mitogenic factors, since conditioned medium (neat or diluted) from cultured ACS isolates enhanced the proliferation of both immortalised (HaCaTa) and, more importantly, primary keratinocyte (NHEK) cultures. Interestingly, these important findings are in contrast to observations from a previous report, where no such positive effects in proliferation was observed for HaCaT cell cultures treated with CM from ACS (Wood et al., 2012). We believe that the lack of enhancement of cell proliferation observed by Wood et al. would be attributable to the use of the original HaCaT cell line. As such cells are cultured in serum-containing medium, the presence of serum could have significantly ‘masked’ any positive effects by the diluted ACS CM.

Human dermal fibroblasts (HDFs) play important roles during would healing, including the responsibility for breaking down the blood clot during healing and producing new ECM structures to support other cells involved in tissue repair. Furthermore, HDFs can differentiate to the phenotypically larger myofibroblasts which express smooth muscle actin (α-SMA). This protein is central in the contractile properties in myofibroblasts to facilitate the mechanics of physically pulling the wound edges together. Typically, when tissue repair is complete, myofibroblasts undergo apoptosis, however if their presence is inappropriately sustained when dysfunctional tissue repair occurs, subsequently causing excess contraction and fibrosis (Penn et al., 2012; Darby et al., 2014). Thus, extensive myofibroblast activity is detrimental to optimal wound healing, leading to scarring and overall poorer healing aesthetics for patients. Furthermore, TGF-beta is abnormally expressed in instances of delayed wound healing, resulting in higher myofibroblast activity, inhibition of fibroblast proliferation, excessive laying of extracellular matrix and higher tension during contraction (Goldberg et al., 2002). It was therefore of interest to investigate whether the application of ACS CM on a wound could affect myofibroblast homeostasis. Using a well-characterised *in vitro* model of HDF differentiation to myofibroblasts, we have provided evidence that our ACS CM regulated the phenotype of HDFs by suppressing the process of cytodifferentiation induced by low density/TGF-β treatment, evident by the reduction in α-SMA expression. Although it remains unclear which specific paracrine factors may have mediated these suppressory effects, clearly the ACS secretome comprises a substantial number of soluble factors, of particular note being EGF and FGF. Previous studies have reported that EGF activity can negatively regulate myofibroblast differentiation via MEK/ERK- and JNK-dependent signalling (Kimura et al., 2013). More importantly, there is extensive evidence for a central role for FGF signalling in curtailing TGF-β induced myofibroblast differentiation, as FGF-2 (known as basic FGF) attenuates scarring during cutaneous wound healing (Spyrou and Naylor, 2002) by suppressing myofibroblast establishment and inducing their apoptosis (Akasaka et al., 2004; Dolivo et al., 2017). The suppressory effects of FGF signalling on TGF-β mediated transition of fibroblasts to myofibroblasts is also supported in the context of disease, such as cancer (Akatsu et al., 2019). Notably, our ACS secretome studies provided evidence for marked increase in FGF-2/FGF-basic expression by Day 3 post culture and, strikingly, it was ACS CM from Day 3 cultures which was used in our experiments (whilst Day 1 culture ACS CM did not show such effects–data not shown). Thus, it is tempting to speculate that suppression of myofibroblast differentiation by our ACS isolates is FGF-2 mediated, however functional FGF-2 inactivation studies would be required to confirm this scenario. Yet, irrespective of the soluble effector mediating these effects, our observations have provided strong evidence that in addition to enhancing keratinocyte proliferation, the ACS-derived cells can attenuate myofibroblast overactivation thus potentially enhancing not only the speed but also the ‘quality’ of wound healing.

**FIGURE 6:**
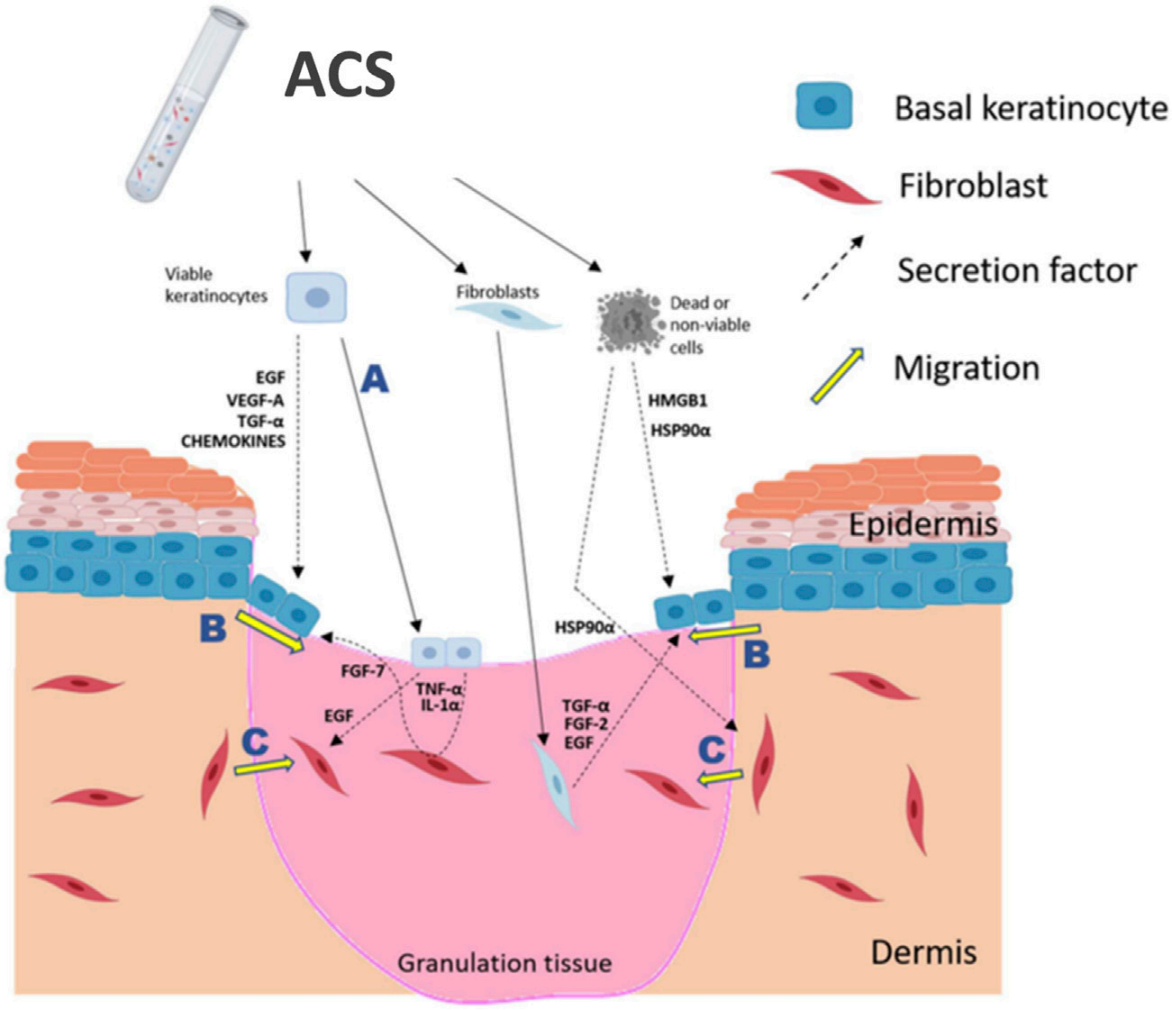
Schematic summary of proposed positive effects of ACS application onto a wound. ACS isolates contain the main skin cell sub-types, such as keratinocytes and fibroblasts (melanocytes are not indicated), as well as non-viable cells which may release DAMPs, such as HSP90α and HMGB1. It is postulated that the rapidly proliferating keratinocytes would be able to “take” into the wound bed **(A)** and thus directly contribute to re-epithelialisation. Keratinocyte activation and migration at the ‘wound edge’ **(B)** would be enhanced by the growth factors, cytokines and DAMPs secreted by cells contained in the ACS. Fibroblast migration into the wound bed **(C)** would also be stimulated by the secreted EGF and HSP90α in order to facilitate HDF-mediated regeneration of the appropriate supporting ECM. The diagram was created using BioRender (www.biorender.com).

In conclusion, based on the recent establishment of our novel methodology for the generation of ACS from split-thickness skin grafts, our study has systematically investigated the sub-populations of cells in the ACS isolates and their biological properties, which collectively are expected to be beneficial upon application to a wound (Figure 6). We demonstrate several important wound-beneficial features including presence of vital cell populations as well as positive modulation of critical steps in wound response (keratinocyte proliferation and suppression of myofibroblasts), and therefore our findings merit further investigation by *in vivo* studies to demonstrate wound benefit. Nevertheless, our work has provided several lines of evidence that the ACS isolates established not only comprise highly viable cell populations that can physically support wound healing but also possess biological properties that have the potential to enhance wound healing, and thus to compensate for the deficiency in essential elements (both viable cells and soluble factors) that are absent in non-healing wounds.

## Data Availability

The original contributions presented in the study are included in the article/Supplementary Material, further inquiries can be directed to the corresponding author.
