# Supplementary figures and images for: A novel method for the establishment of autologous skin cell suspensions: characterisation of cellular sub-populations, epidermal stem cell content and wound response-enhancing biological properties
Source: Front Bioeng Biotechnol. 2024 Apr 5;12:1386896. doi: 10.3389/fbioe.2024.1386896 (PMC11026634; doi:10.3389/fbioe.2024.1386896)

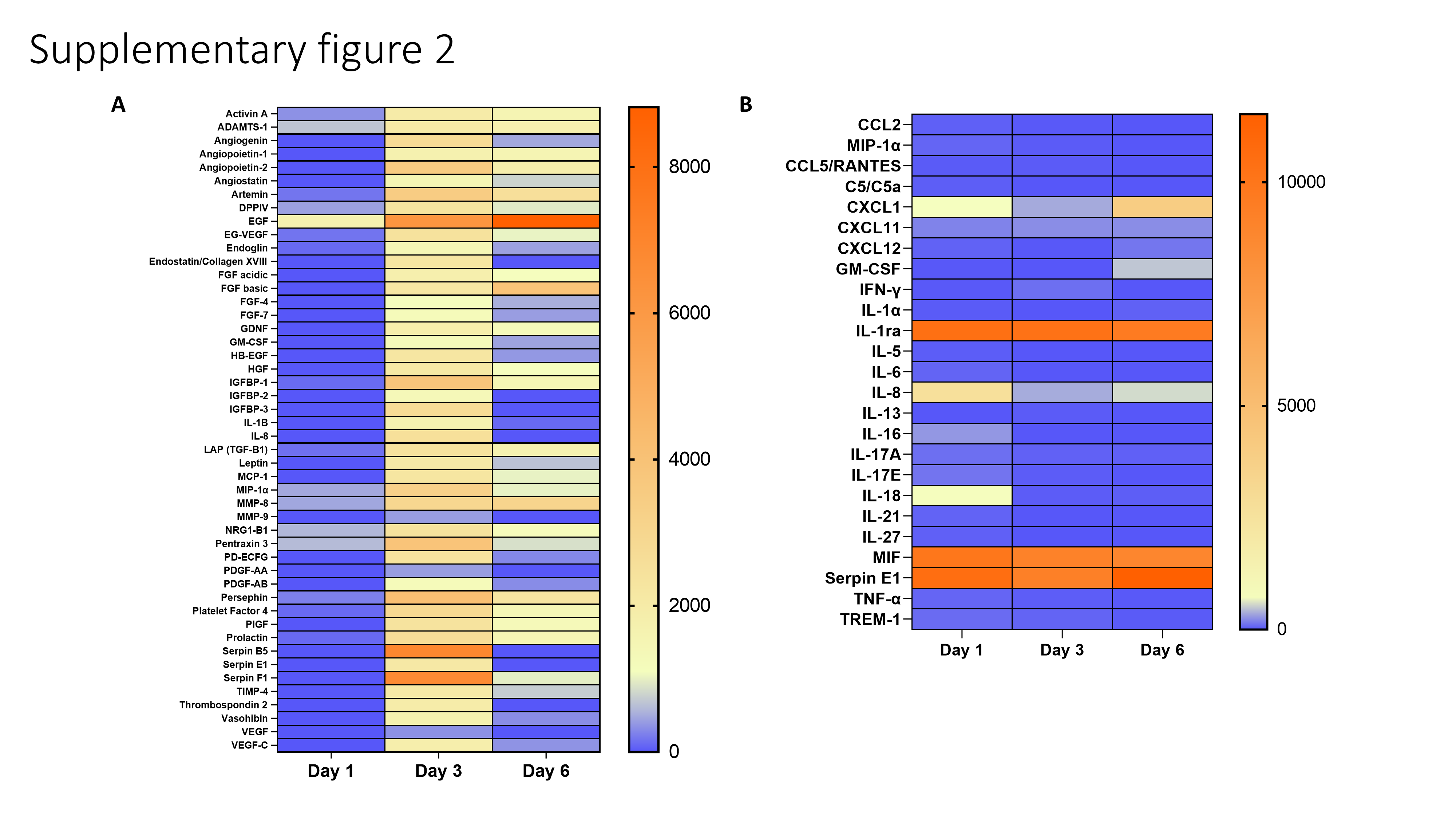

Supplement: Supplementary file 1 [file Image2.TIF]

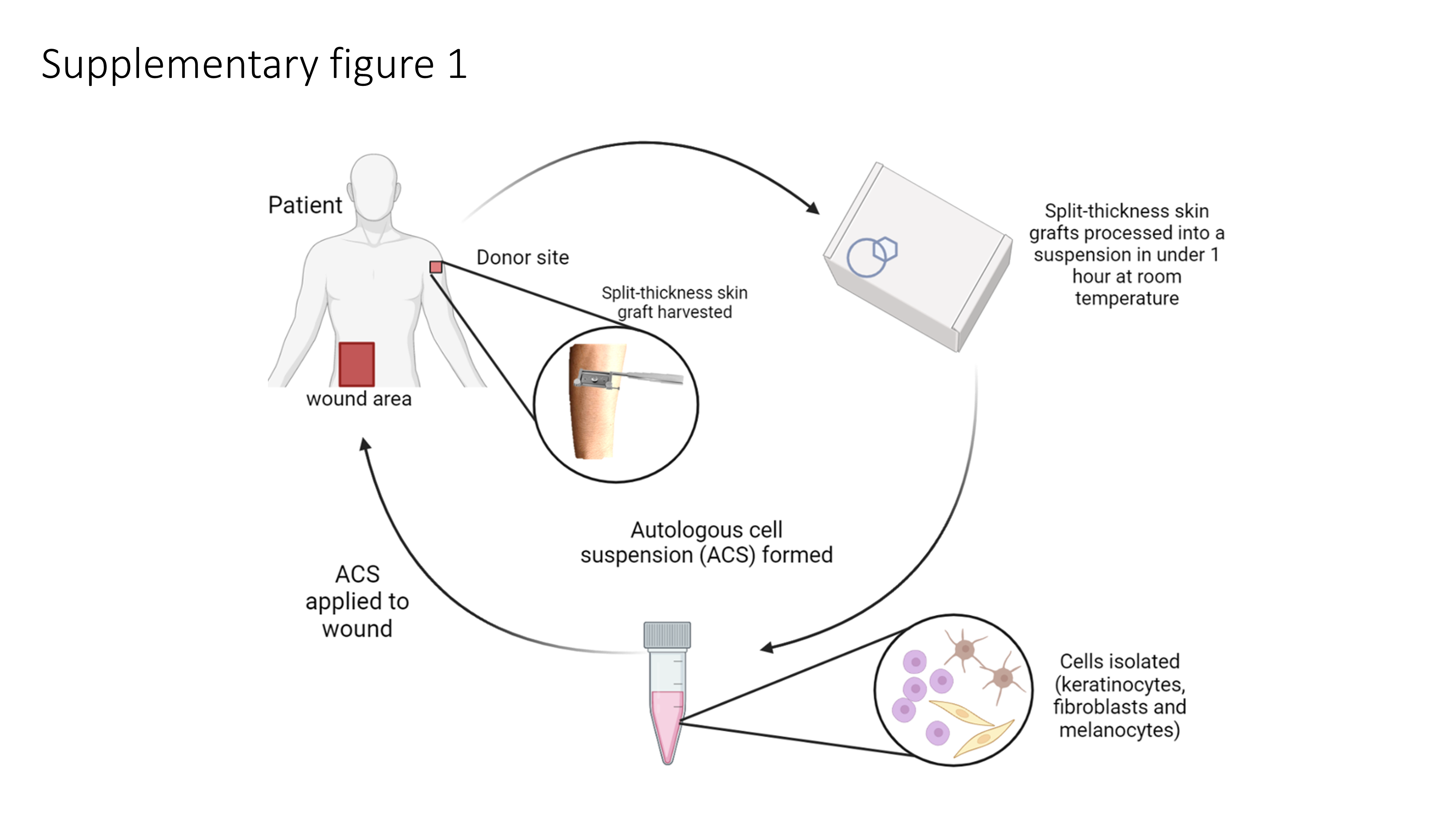

Supplement: Supplementary file 2 [file Image1.TIF]
