# Supplementary material for: A novel method for the establishment of autologous skin cell suspensions: characterisation of cellular sub-populations, epidermal stem cell content and wound response-enhancing biological properties
Source: Front Bioeng Biotechnol. 2024 Apr 5;12:1386896. doi: 10.3389/fbioe.2024.1386896 (PMC11026634; doi:10.3389/fbioe.2024.1386896)
Supplement: Supplementary file 3 [file DataSheet1.docx]

Supplementary Material

**Supplementary figure captions**

**Figure S1.** Schematic summary of the ACS methodology investigated in this study.

A dermatome is used to harvest skin from a patient’s own donor site (which is a relatively smaller area compared to conventional methods) at a thickness of ~0.2-0.3mm and temporarily kept in buffer. The collected skin is subsequently cut into ~2mm^2^ pieces, these are transferred out of the buffer, and placed into a strainer device (of defined pore size) that also comprises a connector ring (for application of negative pressure) that is fitted on a collection tube. 5ml of recombinant enzyme solution (at optimized concentration) is added to the strainer to submerge the skin pieces for 20 minutes. By attaching a syringe to the combined strainer and connector ring, negative pressure can then be applied to remove the enzymatic solution from the skin and into the collection tube. Skin pieces are kept in the strainer for a further 5-10 minutes while sporadically “agitated” using forceps. A volume of 2ml of buffer is then added to the skin pieces (to further dilute the enzyme) and using this volume the skin fragments are vigorously mixed until homogenized. This homogenized suspension held within the strainer and connector ring is then detached from the collection tube and attached to a fresh tube, and negative pressure via a syringe is then applied to collect the ACS. The procedure is repeated twice to ensure maximum cell recovery. Overall, the process permits isolation of keratinocytes, fibroblasts and melanocytes from the donor tissue within <1 hour. Once the above procedure is complete, the freshly-isolated ACS sample can then be applied directly to the wound area, whereby it will accelerate the process of re-epithelialisation and increase efficiency in wound healing completion. The diagram was created using BioRender (www.biorender.com).

**Figure S2.** Proteomic analysis of the ACS secretome.

Semiquantitative analysis presented in the form of heatmap of all soluble mediators secreted by ACS-derived cell populations cultured in serum-free medium at the indicated time points. Supernatants from ACS-derived cultures were collected and analysed as detailed in the Methods. **A)** Relative intensity values of densitometric analysis of ACS-secreted soluble factors using an angiogenesis array. **B)** Relative intensity values of similar analysis using a cytokine proteome array on the same time-points post-isolation. Heat maps were generated using GraphPad Prism (https://www.graphpad.com/).
